# The voices of lived experience: reflections from citizen team members in a long-term care research program

**DOI:** 10.1186/s40900-021-00312-y

**Published:** 2021-10-02

**Authors:** Jim Mann, Roberta Bishop, Graham Bond, Faye Forbes, Barbara Kieloch, Christine Thelker, Stephanie A. Chamberlain

**Affiliations:** 1 Translating Research in Elder Care (TREC) Research Program, Level 3, Edmonton Clinic Health Academy, Faculty of Nursing, Voices Of (potential) Incoming residents, Caregivers Educating uS (VOICES), University of Alberta, 11405 87 Ave, Edmonton, AB T6G 1C9 Canada; 2 Department of Family Medicine, Faculty of Medicine and Dentistry, University of Alberta, 6-50 University Terrace, Edmonton, AB T6G 2T4 Canada

**Keywords:** Citizen engagement, Patient engagement, Health services research, Engagement science

## Abstract

**Background:**

The Translating Research in Elder Care (TREC) program is a partnered health services research team that aims to improve the quality of care and quality of life for residents and quality of worklife for staff in nursing homes. The TREC team undertook several activities to enhance the collaboration between the academic researchers and us, the citizen members. Known as VOICES (Voice Of (potential) Incoming residents, Caregivers Educating uS) we aim to share our experience working with a large research team.

**Methods:**

We reflect on the findings reported in the paper by Chamberlain et al. (2021). They described the findings from two surveys (May 2018, July 2019) that were completed by TREC team members (researchers, trainees, staff, decision-makers, citizens). The survey questions asked about the respondents’ experience with citizen engagement, their perceptions of the benefits and challenges of citizen engagement, and their unmet needs for training.

**Results:**

The paper reported on the survey findings from all the survey respondents (research team, decision-makers, citizens), but much of the results focused on the researcher perspective. They reported that respondents believed that citizen engagement was a benefit to their research but noted many challenges. While we appreciate the researchers’ positive perceptions of citizen engagement, much work remains to fully integrate us into all stages of the research. We offer our reflections and suggestions for how to work with citizen members and identify areas for more training and support.

**Conclusions:**

Despite the increased interest in citizen engagement, we feel there is a lack of understanding and support to truly integrate non-academic team members on research teams. We hope the discussion in this commentary identifies specific areas that need to be addressed to support the continued engagement of citizens and show how the lived experience can bring value to research teams.

## Plain English Summary

Engaging people with lived experience on research teams is becoming more of an expectation for researchers. Published research on this type of engaged research often focuses on the researcher experience with partnered research and neglects the citizen perspective. We are the citizen members of the Translating Research in Elder Care (TREC) program of research, a research program focused on older adults living in nursing homes. Known as VOICES (Voice Of (potential) Incoming residents, Caregivers Educating uS) we aim to share our experiences of working within a large research team. In this commentary we reflect on the pitfalls and opportunities for citizen engagement and reflect on findings from a recent paper published by the research team on the experience of citizen engagement over time.

*If I could be you, if you could be me*

*For just one hour, if we could find a way*

*To get inside each other’s mind*

*Walk a mile in my shoes*

*Just walk a mile in my shoes* (Songwriter: Joe South; Singer: Elvis Presley).

## Background

Since 2011 and the launch of Canada’s Strategy for Patient-Oriented Research (SPOR) [1], many researchers have been tasked with engaging citizens/people with lived experience/patients in meaningful ways that enhance and not hinder the research process. To make engagement successful, new and creative ways of communication, grant application development and project collaboration have and continue to be developed and implemented, often in real time. It was tough slogging and for some researchers, citizen-oriented research still is. The Strategy attempts to incorporate that walk-a-mile-in-my-shoe reality, putting lived experience into research focusing on patient priorities and improving patient outcomes [2].

The Translating Research in Elder Care (TREC) team is a multidisciplinary applied health services research team that focuses on ways to improve the care of older adults living in long-term care homes [3, 4]. In 2015, the team believed there were benefits to including the lived experience voice into their research and created a small advisory group of four members for a specific grant proposal. From there, the plans for this budding advisory group grew and evolved [4].

By early 2016, our group had grown to 11 members and became known as VOICES (Voice Of (potential) Incoming residents, Caregivers Educating uS) [4]. Originally, VOICES was guided by a Terms of Reference that outlined a “consultative and advisory role, when needed”, but it was not long till there were discussions on how to integrate VOICES members more fully into TREC’s many research activities. At this point, members of the TREC team worked with VOICES members to identify priorities for deepening engagement, which ultimately led to a series of activities including a day-long team training on patient engagement and an internal priority setting [4]. These actions, as well as others, took place over a 15-month period. In their paper, Chamberlain et al. describe the results of the TREC team surveys that were done at the beginning and end of that 15-month period [5]. Much of what they report comes from a research and researcher perspective. In this commentary, we highlight findings and direct quotes from Chamberlain et al. [5] and add our perspective on these findings and our experiences as VOICES members over the same period.

## Becoming engaged

Moving from a review-and-provide-feedback model, as originally conceptualized, to more integrated roles has proven to be a bigger challenge than everyone imagined.

While VOICES members were buoyed to see in the survey results that researchers consider our input to be beyond “review and provide feedback” and to include,for example: expanding understanding of the caregiver experience, sensitising researchers to the reality of living with dementia and living in long-term care, identifying ways to make findings relevant, keeping a study grounded, and helping to identify where research is needed [5], we can all recognize that putting these beliefs into action through research can be difficult. Some of the main challenges, as discussed by Chamberlain et al. [5], included learning how to connect individual researchers and VOICES members for specific projects in a way that gave everyone a chance to participate without overloading a few VOICES members, ensuring that VOICES members had time to speak (and respond to others) at large team meetings, and developing the communication tools necessary to keep VOICES members not just informed of what has been done but also to enable them to have a say in decision making and discussions. It has taken time to see these changes – but they are far from fully addressed and still require attention from all team members to keep progress moving forward.

## Roles and expectations

It seems evident from Chamberlain et al.’s findings that *formal* identification of the roles and expectations of VOICES as a group and as individual members can still be a difficult task for both researchers and VOICES members [5]. As described earlier, VOICES was originally created as an advisory group and in many ways, is often still treated as such when it comes to overall functioning of TREC. For example, the described role of the VOICES Committee in the most current Terms of Reference has not changed from the version written in 2016 and still focuses on VOICES’ advisory capacity. So, while VOICES members have come to expect and many TREC members perceive an increase in meaningful engagement based on the results of the survey, the official role of VOICES has not been changed to keep with these expectations. This might seem like unnecessary bureaucracy, but formal recognition of VOICES members and their various roles will be important in moving TREC’s engagement plans forward and ensuring that the promise of meaningful engagement is sustainable. Members of the VOICES team have experience working with other research projects [6] and when asked to reflect on how to address these role-related tensions posited that the organizational structure was perhaps hindering progress. TREC is a large program of research that encompasses many related but distinct projects. The rather amorphous structure of a program of research versus a standalone project has posed both operational and conceptual challenges for VOICES. It may be natural to see an evolution from an advisory committee to more integrated team members on individual projects in cases like TREC and VOICES where there is an interest in both sides in having the citizen engagement in regular research activities; on the other hand, perhaps more clarity is required on the parameters of an advisory committee, if they are to maintain that function over the long term.

Chamberlain et al.’s follow-up survey results indicate that over the 15-month period, researchers believed that VOICES members should have an expanded role throughout the research process beyond simply advisory, including “soliciting VOICES expertise before a grant is proposed to ensure that their interests shape the research questions,” yet the researchers described many practical issues actually putting this belief into practice [5]. Issues included short grant timelines (i.e., from when an opportunity is announced to when it must be submitted), feeling insecure in their written and verbal communication skills with non-academics, and needing meeting facilitation support. These practical concerns are understandable and as non-academics we have come to appreciate the time pressures and deadlines that the team are under. However, rather than continue to describe how we still struggle for better integration perhaps we need to consider these integration efforts from a broader focus of team improvement. What can we all do to support each other? We urge the TREC team and others to simply consider how they might reach out to any new team member and how they would build a working relationship, irrespective of their citizen status. We understand that there are differences in our experience and familiarity with the research process, but it is an important starting point. VOICES members do not expect perfection and while attention towards non-academic communication skills and meeting facilitation are important, we do not want the search for perfection to hinder our participation. While it may not be ideal, reaching out and requesting our attendance at a meeting, providing materials with the caveat that you are under a deadline and details are not complete, as you might with an academic team member, moves us towards a more realistic partnership.

## Tokenism

*Managing expectations, avoiding tokenism, adequately identifying and accommodating citizens’ needs* (Researcher, follow-up survey).

Engagement of citizen researchers is challenging for citizen members if expectations are not explicit, and engagement is sometimes peripheral. Concerns of tokenism are also noted among VOICES members. Anecdotally, there is concern of not being taken seriously and to be merely a check mark on a grant application form or a voice at a team meeting but not having been actively involved in the work being presented; or that we will be invited to participate and then find our participation is not consistent or is diminished. Findings from the surveys reflect this tension—that the team want to involve citizens but continue to struggle with the practical steps and lack preparation for the full scope of actual engagement. This continues to be a work in progress for many within (and outside of) TREC. As Jim Mann noted in the text *Everyday Citizenship and People with Dementia*, “An individual who has made the commitment to collaborate on a research project has done so believing they will be respected and their input valued. [To do otherwise] would be disrespectful and counter to the search for robust outcomes [7].” Moving away from tokenism to integration means that program and project leads need to be clear with their citizen partners about the role and the associated activities. Roles and expectations may change, as evidenced in our shift from advisory committee to team members, but true integration means respecting your citizen partners enough to be in regular communication with your members about the scope of their role and its limitations. Another area for team improvement, not just from the citizen perspective, is to have project leads provide a timeline of project activities and how members can be included in these activities. We may not be involved in every stage of the research but not being made aware of the ongoing work or any delays keeps us on the outside, does not give us the opportunity to engage in the work, and does not suggest we are valued by the team. Finally, rather than be concerned about if/when to include citizen partners on team meetings, ask yourself if you would include other partners? If the answer is yes, we should be included and moving forward, included on all of the team meetings and other communications.

Tokenism and issues of representation extend to citizen advisory committees. VOICES continues to grapple with diversity. VOICES includes members from across Canada, including urban, rural, and remote regions and an approximately equal gender split. During VOICES’ expansion in2016, we did not purposefully seek representation from minority groups except the LGTBQ community (because of connections with a local advocacy group) and so do not have members from immigrant, non-White, or non-Christian communities. While VOICES has agreed to address diversity going more purposefully forward, questions have been raised about how that will look in practice, given that much of the data TREC analyses comes from routinely collected health data in LTC homes that does not capture information on race, ethnicity, or other critical identity factors.

## Working together and communication

*Current attitude/aptitude of many researchers is a major barrier* (Staff, baseline).

Adapting to the notion of co-research with a citizen partner takes time and reflection. We recognize that researchers may question how they acknowledge the central importance of lived experience while not discounting their own expertise [7]. At the same time, VOICES members also must reflect on their possible contributions to research. Some in our VOICES group have questioned their own capability in a co-researcher role but, on reflection, see the value their lived experience brings as a team member to specific projects.

Communication is key. We have learned that we need the researchers to provide a clear introduction to the research vision and an idea of how citizen partners fit in. The VOICES members, through this process, will have the opportunity to determine their interest and suitability to the project and offer their thoughts on their role. Much like an informal interview, both parties are able to look for a fit between the researcher’s needs and the co-researchers’ lived experiences past and present. Taking the time at the very start will set the stage for a collaboration that is based on mutual trust and respect [8]. During one of our early VOICES meetings, we all wrote down our previous experiences and highlighted skills we felt could be useful in the research program (e.g., business and management, on-air television producing). This exercise demonstrated not only our experience as persons with a dementia and caregivers but our other skills that might have been overlooked if we were just focusing on our roles as VOICES members. This exercise and efforts to highlight our array of skills as a group have proven to be essential at various phases of research.

*Concern when a small group of citizen partners are key consultants on multiple projects, both in terms of fatigue and rigor* (Researcher, baseline)

Many researchers expressed concern, here and elsewhere, about partner fatigue and overuse but, really, this is something that only the partners can speak to [9]. We encourage researchers everywhere to not pre-judge, presume, or pre-determine. As Mann & Hung highlighted in their paper, “Jim taught me the meaning of ‘burden’, like risk and benefit, is subjective as *it depends on people’s values and beliefs*”[8]. If there is a job for the citizen member to do, inquire. Don’t assume they are too busy or lack interest. If there is a meeting to attend related to the research, don’t presume to know the member would not wish to attend. Ask. It is important that researchers not let their own concerns about burdening citizen partners interfere with the opportunity for fulfilling engagement. At the same time, all team members – researchers and partners – need to recognize the resources required to support good communication. As Chamberlain et al. note in their paper having a staff member or other champion to ensure that communication standards are upheld is important to making engagement work for everyone.

## Measurement—the proof is in the pudding?

Is it enough to say citizen engagement benefits the research making it “more meaningful and relevant” and gives the researcher “greater authenticity or relevance,” as reported in Chamberlain et al.’s paper, and if it is, how does citizen engagement become standard practice and how is that sustained? Chamberlain et al.’s paper describes team training that included VOICES members, researchers, staff, and other knowledge users. We began the team training optimistic that we would learn new skills and techniques to advance the team’s engagement however we found that it provided only a superficial overview of citizen-oriented/patient-oriented research and the ways that citizens could be engaged. The training did not address the practical concerns that we have highlighted. Perhaps most concerning was that it did not identify ways to continually monitor engagement, a particular issue in a large program of research such as TREC. It is not just for researchers but for citizen partners that this is important. How do we measure and report on how often VOICES partners are enlisted in projects, at what stage is their input sought, and how is that input incorporated into the research? VOICES members and other citizen partners need to hear regularly from their researcher counterparts. Finding ways to measure all these aspects may help us better understand if researchers are appropriately drawing on the unique perspectives and knowledge built from their partners’ lived experience – and if not, how we can continue to work towards that goal together.

## Conclusion

Chamberlain et al.’s paper shows TREC researchers value involving citizen partners throughout the research process and the experiences to date, including challenges to engagement [5]. For their part, VOICES members have expressed a range of experiences from a sense of appreciation for being consulted to frustration at not feeling fully engaged by researchers at all stages of the research process.

Perhaps the paper and our Commentary will launch a review of how we discuss and support engagement both within TREC and elsewhere. Both pieces highlight the need for early and meaningful engagement and for appropriate metrics of the extent to which teams are meeting their engagement goals. Externally, we need to see more training, practical tips, and support for engagement that puts the value of lived experience on par with the value of research experience.

VOICES is not unique as a lived experience group of advisors asking questions and seeking a clarification of its role. For example, Mann & Hung developed *ASK ME*, practical tips when doing co-research with people with dementia, but has relevance to the engagement of citizen-researchers with lived experience [8]. As this Commentary has illustrated, there are challenges on both ends when including citizens with lived experience in research projects but they are not insurmountable.

## Data Availability

The data from the generated in the study on which this commentary is based are available from the manuscript’s corresponding author on reasonable request.

## Abbreviations

TREC: Translating Research in Elder Care
VOICES: Voices Of Individuals, family and friend Caregivers Educating uS
